# The Magnitude and Impact of Bullying among School Pupils in Muscat, Oman: A Cross-Sectional Study

**DOI:** 10.1155/2014/169737

**Published:** 2014-10-14

**Authors:** Muna Al-Saadoon, Yahya M. Al-Farsi, Syed Rizvi, Marwan Al-Sharbati, Abdullah Al-Jabri, Sufyan Almamari, Wafaa Al-Baluki, Samir Al-Adawi

**Affiliations:** ^1^Department of Child Health, College of Medicine and Health Sciences, Sultan Qaboos University, Muscat, Oman; ^2^Department of Family Medicine and Public Health, College of Medicine and Health Sciences, Sultan Qaboos University, Muscat, Oman; ^3^Department of Epidemiology, School of Public Health, Boston University, MA, USA; ^4^Department of Behavioral Medicine, College of Medicine and Health Sciences, Sultan Qaboos University, P.O. Box 35, Al-Khoudh, 123 Muscat, Oman; ^5^College of Medicine and Health Sciences, Sultan Qaboos University, Muscat, Oman

## Abstract

Research about bullying among school pupils in the Arab/Muslim population is scarce. This study evaluates the characteristics of bullying and its impact among school pupils in Oman via cross-sectional survey among eighth grade school pupils (*n* = 1,229) during the academic year 2006-2007. The participants were selected using stratified random selection among 6 administrative divisions of one the governorates in the country. Data were collected using self-completed structured questionnaires. This study found similar percentages of males and females (76%) have experienced one form of bullying, and the majority of the incidents (80%) occurred in the vicinity of the school. In almost half of the cases, the bullying was initiated by a student of the same age or older than the victim. The most common type of bullying encountered in this study was verbal (47.7%), followed by misuse (45.9%), physical (43.9%), and, finally, social isolation/exclusion (22.5%). Although the failure of an academic year was uncommon among victims of bullying, the number of pupils who missed 4–6 and ≥7 school days was higher among bullied pupils. If this study will withstand further research, educational initiatives are needed to mitigate the rate of bullying in Oman.

## 1. Introduction

Various studies have attempted to determine the common stressors that have a persistent and pervasive impact on the development of children [1, 2]. Among the varied forms of stress that children can often fall vulnerable to at their tender age is being bullied at school [3]. Bullying, or behaviour intended to cause an adverse effect such as physical harm or mental distress to others [4–6], has been commonly observed among children ranging across different ethnic and cross-cultural groups [7, 8].

Bullying has been compared across nations. Craig et al. [9] have examined the rates of bullying and victimisation in 40 countries across the globe among nationally representative pupils aged between 11 and 15 years (*n* = 29,127). These countries generally fall under the category of middle- to high-income countries. The study unequivocally showed that exposure to bullying is a global problem, with the incidence ranging from approximately 9 to 45% among males and 5 to 36% among females. Other studies have reached similar conclusion [10, 11]. However, an international comparison of bullying rates is hampered by two main inconsistencies: the fact that the term “bullying” lacks universal defining features and that there are variations in the methodologies employed to detect incidences of bullying. Nonetheless, in the countries where surveys have been conducted, despite the variety of methodologies and the lack of a standardised concept of bullying, the rate of bullying among school pupils appears to be remarkably high. Studies indicate that the prevalence of being bullied among school pupilsin North America ranges from 40 to 80% [12]. There is some indication that incidents of bullying appear to be declining among Euro-American populations [13]. In India, Kshirsagar et al. [14] recorded that approximately 30% of the children interviewed in their study reported having been bullied. In South Korea, another Asian country, the rate of various forms of bullying ranges from 5 to 12% in the population surveyed [15]. Siziya et al. [11] examined the prevalence of bullying among a nationally representative sample of adolescents in grades 7 to 10 in an African population in Zambia. The study reported that more than 60% of these children endorsed the view that they had been bullied over the previous 30 days.

Examining the continuity of bullying over time is also important. In a longitudinal study conducted in Canada, Beran [16] explored a concept known as “the stability of harassment” in children. The data from this study indicate that those who were not initially bullied had less chance of being bullied in subsequent years. In contrast, the children who had previously been targets of bullying were more prone to experience bullying later. Other studies are consistent with this view [17]. There are empirical studies suggesting that the repercussions of being bullied for school pupilsare enormous, including potentially triggering the onset of mental disorders, which can in turn have negative ramifications on quality of life. There is also evidence that the psychological effects of bullying persist for many years after the actual incidence of bullying, with variability among victims depending on the social support provided for them [18]. Long-term self-reported antisocial behaviour, such as delinquency, violence, and drug use, was found to be related to bullying. Victimisation has also been associated with suicidal ideation, and this association was stronger for those adolescents who were bullied regularly on a weekly basis [19]. Although there is a strong belief that bullying has a significant impact on academic achievement, it is still unclear whether academic underachievement stems from scholastic failure or bullying-related absenteeism [20].

To our knowledge, with a few exceptions, minimal research exists in the literature to reflect the magnitude of bullying within the Arab/Islamic region. In Turkey, Arslan et al. surveyed pupils in the ninth and tenth grades of 6 high schools in the Istanbul province during the spring of 2007 and reported that 17% of the students admitted to having been bullied [21]. Gofin and Avitzour examined the rate of traditional bullying among Arab and Israeli students in Jerusalem in 2006, and traditional bullying was found among nearly 15% of these students [22]. Oman represents a fertile ground to explore the magnitude of bullying and its impact on indices of education engagement. In 2005, a brief study in Oman explored the bullying prevalence and demographics. The study showed that 40% of middle-school students in Oman had experienced bullying in the past 30 days [23]. However, little is known regarding the type and impact of bullying in Oman. Along with such questions, research exploring the places of abode or the educational settings is also imperative, as previous studies have alluded to the importance of differentiating areas of residence in determining factors of bullying [24]. In demographic terms, the Omani population is characterised as currently in a “youth bulge” phase, meaning that the majority of the population are children and adolescents [25]; this is the age group most widely acknowledged to be vulnerable to bullying. This gives a strong rationale for exploring the type and impact of bullying among emerging Oman's “youth bulge” population.

To our knowledge, no prior study has been undertaken to examine the magnitude and other correlates of bullying in Oman. This study aims to explore the incidents, characteristics, and impact of bullying among eighth grade pupils in Muscat Governorate of Oman.

## 2. Methods

### 2.1. Participants

The study was conducted during the period from August 2006 to February 2007 in the Muscat Governorate, a region of Oman including the coterminous capital city of Muscat. The governorate is divided into 6 administrative divisionsor* wilayats*: Al Amerat, Bawshar, Muscat, Mutrah, Qurayyat, and As Seeb. All participants from the selected schools were invited to participate in the study during the academic year 2006-2007.

According to statistics available from the Ministry of Education, in the academic year 2006-2007 there were 101,597 pupils in the Muscat Governorate enrolled in public schools (which are schools run by the state under the direction of the Ministry of Education). In total, there were 49,783 (49%) male and 51,814 (51%) female students. For the present purpose, all participating pupils were children who were enrolled in the eighth grade. The sample size was estimated using the EPI Info statistical program Version 6.0 (Centers for Disease Control and Prevention, Atlanta, Georgia, USA) and based on the formula for calculating sample size in cross-sectional studies as elaborated by Kelsey et al. [26]. With a type-1 error of 5% (*α* = 0.05) and 95% level of significance, it was estimated that 1,153 subjects would be required in order to detect a bullying prevalence as low as 25% at a power level of 90%. Therefore, the set target was to reach 1,153 participants in order to achieve the objectives of the study.

The participants were selected using stratified random selection. Schools in the catchment area were randomly selected with an equal representation of both genders. The second stage of the sampling was done by randomly selecting one of the eighth grade classes in the school and inviting all of the students in that class to participate in the study. All students were given invitation letters addressed to their parents/guardians describing the rationale for the study. The parents/guardians were asked to sign consent forms if they consent to the study. In addition to receiving parental consent, the pupils were also required to give consent before taking part in the study. The invitation was sent to 1500 students, of whom 1263 responded giving a response rate of 84%. Furthermore, 34 students were excluded due to missing sociodemographic data or incomplete segments of questionnaire. Finally, a total of 1229 students were included in the analysis, of whom female students constituted 55%.

### 2.2. Instruments

The data were collected* via* a self-completed questionnaire specifically developed for the study using the available literature. The instrument was structured in a Likert questionnaire item, whereby respondents were asked to specify their level of agreement or disagreement on a symmetric agree-disagree scale for a series of statements. Questions were formed to cover the different attributes and characteristics of bullying that manifest as “verbal,” “misuse,” “physical,” or “social,” over the period of the last 12 months.* Verbal* bullying is operationalized here as statements that are perceived as threatening because of their negative connotations (“*I was teased, mean things were said to me, I was called name, I was threatened*”).* Misuse* is defined as bullying that falls under the category of interpersonal exploitation (“*Things were taken from me, my property was damaged, etc.*”).* Physical* bullying entails hurting a person's body or possessions (“*I was hit, kicked, pushed, slapped, spat on, etc.*”).* Social* bullying, also known as* relational* bullying, involves negatively affecting someone's reputation or relationships (“*I was excluded, ignored, had rumours spread, mean things said about me to others, others were made not to like me, etc.*”). The draft questionnaire was circulated to experts in the country who were likely to be well versed on issues pertinent to school bullying. After protracted discussion during a focus group, 10 items were deemed to be the most suitable and socioculturally relevant for investigating bullying in Oman.

The final section of the questionnaire covered demographic information including age, gender, and number of siblings as well as the birth order of the participants. In addition, some relevant information pertinent to education and performance was also sought from the students themselves.

### 2.3. Procedure

The questionnaire was piloted on 50 pupils (25 males and 25 females). Following the pilot phase, a few changes to the questionnaire were made for clarity and comprehensibility. Also, it was decided that one member of the research team would read the questionnaires aloud to the class and that a period of time would be allotted after each question for the participants to write/select their answers. This method was adopted to ensure equal understanding of the questions among all included pupils, as reading ability was found to be variable among the students as deduced from the varying times taken to complete each questionnaire.

In the pilot study, the validity of the questionnaire was assessed by comparing the information obtained through self-completed questionnaires versus questionnaires filled out during interviews of all the 50 pupils included in the piloting. Therefore, all pupils were asked to complete the questionnaire by themselves, and later they were asked to fill out the questionnaire during the interviews. The information gathered using the questionnaire filled during the interview was considered the “gold standard,” to which the self-completed questionnaire was compared. Convergent validity was assessed using Spearman's correlations over the total bullying frequencies (*r* = 0.83, *P* < 0.01) which showed significant correlation, supporting the good convergent validity. Interrater reliability was established among pupils at a standard of 90% agreement on key questions related to bullying in the questionnaires filled during interview and the self-completed ones. The average percentage agreement between the pupil performance in both questionnaires (self-completed and during interview) was found to be 91%. The interrater reliability of questions related to bullying frequency and characteristics was also assessed using Kappa coefficient as a parameter which was found to be 0.82, indicating high reliability. Combining all psychometric parameters, the global psychometric assessment of the questionnaire indicated high reliability and validity.

The data were collected by three of the authors after receiving training on data collection* via* interviews. All attempts were made to adhere to the World Medical Association's “Declaration of Helsinki” for ethical human research, which encompasses confidentiality and data storage [27]. In brief, the pupils were explicitly assured that their participation was anonymous and voluntary, that the data gathered would be aggregated, and that they could withdraw from the study at any time, without prejudice. In the event that undue distress was experienced by the pupil while responding to sensitive questions, counselling support was offered if needed. The pupils were also asked not to discuss the questions among themselves in order to avoid peer influence.

### 2.4. Data Analysis

Chi-square analysis was used to evaluate the statistically significant differences between the proportions of categorical data. The nonparametric Fisher exact test (two-tailed) replaced the Chi-squared test in cases of small sample size where the expected frequency was less than 5 in any of the cells in the 2 × 2 tables.

Statistical analysis was performed using the Statistical Package for the Social Sciences (SPSS) software Version 19.0 (IBM Corp., Chicago, IL, USA); a cut-off value of *P* < 0.05 was considered the threshold of statistical significance for all tests.

## 3. Results

A total of 1,229 pupils were enrolled. In terms of breakdown of gender, approximately 45% were male (*n* = 554) and 55% were female (*n* = 675). The differential proportions of males and females were due to differential response rates among the two groups.  Table 1 shows the sociodemographic characteristics of the study participants. The majority were 13- and 14-year-olds attending the eighth grade. There was an adequate representation of all the* wilayats* in the Muscat Governorate region. In total, 940 participants (76.5%) reported that they had been bullied, and bullying was comparable among females and males (76.7% versus 76.1%, resp.).

Table 2 shows the characteristics of past bullying incidents among the participating pupils. The majority reported a frequency of 1–3 incidents of bullying over the past 12 months. The percentage of pupils who reported 4–10 incidents was 13%, and 14.7% reported that they had been exposed to more than 10 incidents of bullying. The distribution of bullying frequencies was comparable between male and female pupils. The most common type of bullying was verbal, followed by social and then physical. The least reported type was misuse. Compared to the female pupils, verbal and physical bullying were more common among males. However, social and misuse bullying were more common among females. The difference in types of bullying among males and females was statistically significant. The most common site for bullying was reported to be in a school environment, followed by on the bus, in the neighbourhood, at home, and in shopping areas. Bullying on the bus was reported more by males, while females reported more bullying occurring at home. As Oman is a gender-segregated society, such variation might stem from the differences in how each gender spends their time. In Oman, the expectation is that females will remain mostly in the domestic sphere while males are accepted to spend more time in the public sphere. From a cultural perspective, this means that girls are expected to stay at home while the boys are allowed to freely spend time outside in the community.

Table 3 shows the characteristics of the perpetrator of the bullying incident and the victim's reaction towards the incident. The majority reported that the perpetrator was older than them and tended to be a known person. This pattern of age and relationship of the perpetrator to the victim was more prominent among female pupils compared to males, and the differences were statistically significant.

The exact motive for the bullying was unknown to the majority of the victims. Nonetheless, the victim's physical appearance was the most reported reason for the bullying, followed by academic performance and the victim's style of speech. The least reported reason was a victim's disease or disability. Compared to female pupils, male pupils have reported higher proportions of academic performance, victim's style of speech, and victim's disease or disability as reasons for bullying, and the differences were statistically significant. The majority of the victims reported the bullying incident to another person such as a school staff member, a relative, or another student. About one-third of the victims fought back either verbally or physically. However, about one-fifth (19%) of the victims did not take any action against the perpetrator, and the difference between females and males was small (20.3% versus 17.3%, resp.). More female students tended to inform a relative or another student, while more male students tended to inform a school staff. Compared to females, more male pupils fought back physically. The variation in reciprocating the bullying was significantly different between males and females.

Table 4 compares bullied and nonbullied pupils in terms of failed academic years and missed school days. Overall, the proportion of pupils who failed one or more academic years was more common among nonbullied pupils compared to bullied pupils. The differences were statistically significant (*P* = 0.02). On the contrary, missing 4–6 and 7 or more school days was more common among bullied pupils compared to the nonbullied pupils, and the difference was statistically significant (*P* = 0.01).

## 4. Discussion

Oman has been internationally lauded for spending 3.5% of its gross national product (GNP) to furnish a universal free educational system for children, an important springboard for equipping the future generation with the required knowledge and skills to compete in the modern world [28]. Educational institutions have spread to all corners of the country and there is no gender gap in the gross enrolment rates for secondary education. As a result, illiteracy has generally waned among the younger generation. On an educational front, this progress is noteworthy, considering that four decades ago there were only 3 primary schools, and these were reserved solely for male children [29]. However, the present study addresses whether this education is provided in a safe environment in Oman.

The first aim of this study was to explore the characteristics of the bullying incidents among eighth grade pupils in the Muscat Governorate region in Oman. As there is currently no universally accepted definition of bullying, the rate of bullying appears to fluctuate in a complex way. Although the focus is often geared towards the victims of bullying, the presence of bullying perpetrators highlights the presence of antisocial behaviour in society. In either case, this behaviour is likely to burden the society with negative social and economic implications [30].

Based on multicentre studies using the Global School-Based Student Health Survey, Fleming and Jacobsen reported that approximately 40% of middle-school students in Oman had experienced bullying in the past 30 days [23]. The present study suggests that approximately 76% of eighth graders in Oman had been bullied during the previous 12 months. Depending on the definition of what constitutes bullying and the time frame, there are wide variations in the reported rate of bullying among school pupils. The rate of bullying presently found in this study falls within the globally reported rates ranging from 5 to 90% [11, 16, 31]. This study therefore unequivocally suggests that the rate of bullying in Oman is skewed toward the higher end of this range.

Several studies suggest that certain sociocultural teachings, such as those found in “collective societies” or groups that highly value conformity, perceive bullying as an acceptable means to pattern idealised social norms [32, 33]. Nonetheless, evidence from Japan suggests that* Ijime* (school bullying) coincides with the onset of acculturation and modernisation and with the weakening of the traditional bonds that are an integral part of a collectivist society [34]. In the last decades, which have been associated with the development of the oil industry, Oman has undergone a rapid socioeconomic transformation that has resulted in an unprecedented increased standard of living and acculturation. There is indication that such affluence has negative repercussions in denting challenging traditional outlooks that foster collective identity [35]. It remains to be seen whether the presently observed high rate of bullying in Oman stems from the sociocultural trajectory of the traditional to the more recent atmosphere of acculturation and modernisation. Further studies on this subject are therefore imperative.

This study showed that the most common type of bullying was verbal, followed by social and then physical. The available literature is replete with what constitutes bullying. One of the leading authorities in the field of bullying, Olweus, has categorised bullying into 3 types: physical, verbal, and social exclusion [4]. Rivers and Smith have classified bullying incidents as those stemming from direct physical bullying, direct verbal bullying, and indirect bullying [5]. Others have suggested that physical bullying, verbal bullying, isolation, rumour-spreading, and harming are integral parts of bullying behaviour [6]. As there has been no unified definition of bullying, there is an anecdotal and impressionistic observation that bullying among school pupilsin Oman generally falls under generic terms such as verbal and physical, but this is likely to have explicit manifestations such as being subjected to misuse as well as the social ramifications that this entails. For these reasons, the present study classified bullying under the categories of verbal, misuse, physical, and social.

In terms of gender, this study showed that verbal and physical bullying were more common among males compared to females, while social and misuse were the more common bullying types experienced by females. The gender difference in being bullied has been consistently shown in the literature. The commonly reported pattern is that females are more prone to “nonphysical” types of bullying [36, 37]. Conversely, physical bullying appears to be more dominant among males. Studies of traditional societies indicate that prescribed gender roles have a direct bearing on the type of bullying an individual is likely to experience [38]. Thus, studies have suggested that females are likely to be victims of verbal abuse whereas males are likely to affected by physical abuse; this is concordant with the view that “girls manipulate and boys fight” [39].

This study has also attempted to determine the characteristics of bullying incidents and the perpetrators, as well as the victim's reaction towards the bullying. Significant numbers of bullying perpetrators for both genders were noted in the present study to be older than the victims. In agreement with the available literature, it was also found that most perpetrators of bullying tend to be those with whom the victim is familiar, echoing the idiom that “familiarity breeds contempt” [12, 40, 41]. Such situation appears to prevail in Oman.

It has been estimated elsewhere that 8 out of 10 disabled children are bullied and that being bullied is likely to create a substantial psychological burden [42, 43]. From the present study, the link between disability and bullying was not endorsed; this therefore merits some speculation. In Oman, many children with disability are often not sent to school [44, 45]. Therefore, it is possible that there were no participants within the study with overt disabilities. In traditional societies such as Oman, disabilities are equated with the incarnation of or possession by spirits which have capacity to “infect others” [46]. There is also the traditional belief that laughing at or mocking a disabled person will incur bad luck. Therefore, it is possible that individuals with a disability in Oman are spared from being bullied, unlike other countries. More studies are essential to scrutinise this view.

A sizeable quantity of literature exists on the “gut reaction” of youngsters subjected to bullying [47, 48]. As expected, victims who reacted to bullying constituted the minority, as only 37% informed others of the incident and 32% fought back either with a verbal response or by using physical means.

It is apparent in the available literature that there are many negative repercussions to bullying including in the domain of school “engagement/disengagement” [49, 50]. School engagement has been previously defined by Newmann in terms of students making a psychological investment in knowledge acquisition [51]. Accordingly, the learners “try hard to learn what school offers… (and) take pride not simply in earning the formal indicators of success (grades), but in understanding the material and incorporating or internalising it in their lives” [51]. Mehta et al. [52] have examined the hypothesis that bullying has an adverse effect on the variables relevant to school engagement. They unequivocally found that a “bullying climate” tended to breed lower commitment to the school and less involvement in school activities. Such occurrence was independent of the individual's gender, race, or school size or whether they were of an ethnic minority or otherwise [52]. The results of other studies are congruent with this view [53, 54].

To evaluate the impact of bullying on indices of school engagement, this study explored whether indicators of school failure (repeating academic years) and absenteeism (absence from school) were more common among victims of bullying. The underlying assumption is that bullying predicts weakened academic performance* vis-à-vis* the resulting emotional distress and school disengagement. The present data suggest that the tendency for academic failure was more common among nonbullied compared to bullied participants. This finding, at face value, ostensibly discounts the previous view that being bullied is a strong predictor of negative academic performance or the indices of school engagement [43, 53–55], but there is some preliminary evidence that bullying has a more complex relationship with academic performance and that the link may not be a temporal one [56]. Some speculation for the present findings is therefore imperative. The reason that there were a higher percentage of pupils repeating an academic year among the nonbullied may be because the nonbullied included those who were already academically poor students. Moreover, some of them may not have been the victims of bullying but instead been perpetrators. However, investigating the second index of engagement, school absenteeism, indicated that the proportion of participants who missed 4–6 and 7 or more school days was more common among bullied participants compared to the nonbullied.

Various limitations emerging from the present study ought to be highlighted. Firstly, as aforementioned, the items of the questionnaires were drawn from the literature but were specifically chosen to suit the sociocultural context of Oman. By doing so, rather than employing measures with established psychometric properties, this study was limited by the virtue of having employed measures of which the validity and reliability have not yet been established. This limitation has obviously hampered the much-needed establishment of a universal taxonomy of bullying, relevant to international comparisons. In relation to this, despite the plethora of studies on bullying among school pupils, there is little consensus on the validity and reliability of the measures employed. Many authors have appointed the limitations of self-reported measures, such as the self-completed questionnaires used in this study [57]. Related to this, the student's own definition of bullying was also not taken into consideration and this might have affected the reporting of different types of bullying. Another related limitation was that the questions about possible motives for bullying were directed towards the victims, while it would have been more logical if they were directed towards the perpetrators. Future studies ought to be soliciting the review from both victim and perpetrators of school bullying.

Secondly, Oman is a vast country with much subcultural diversity. This study is limited by the fact that it was carried out in the urban capital, Muscat, and its satellite region. On this ground, the generalisations of this study are limited to the urban population. The confidence in establishing a relation between bullying and poor academic performance might be limited by the fact that it has employed a cross-sectional approach. Such research methodology is not equipped to capture cause and effect as there is no mechanism to establish a temporal relationship. In addition, the observed association between being bullied and absence from school might have been affected by another yet to be recognized confounding factor. As a matter of fact, the study did not take into consideration that school absence may have been due to other factors (illness, accidents, outings, etc.). It is possible to assume that the reasons for school absence may have been different among bullied and nonbullied students. Finally, the present study appears to provide a snapshot of the victim of bullying. In future studies, it would be interesting to examine more extensively the characteristics of those who bully others. This could provide an overview of both sides of the bullying coin that would have implications for contemplating evidence-based antibullying curricula.

It is essential here to highlight some implications for school health. This study largely substantiates the view that bullying, or behaviour intended to cause adverse reactions such as physical harm or mental distress to another, transcends geography, ethnicity, or religious denomination [7]. This study supports the view that Oman is not immune to the vagaries of bullying, as the present results indicated that 76% of eighth graders had endured bullying in the last 12 months. Such a substantial percentage appears to be skewed towards the higher end of the range regarding bullying rates, according to the available international trend [11, 16, 31]. In a society known to be characterised by a “culture of silence,” such as in Oman, those affected by bullying are likely to have slipped under the “radar” of concerned parties, and, therefore, bullying has so far remained a furtive activity. It would be worthwhile to note that some bullying also occurs outside the vicinity of the school and that some of it may stem from known person. Therefore, teachers and school authorities ought to be educated in detecting the presence of bullying using culturally sensitive lenses, as well as in providing timely interventions. The curriculum should be specifically designed with the intention of providing education on the impact of bullying. The authorities in the Ministry of Education should employ the mass media at their disposal to combat bullying. Oman, as a society, has been known to thrive on its diversity; such an attitude could be used as the catalyst for prevention and intervention. As 76% of eighth graders have endured and suffered from harassment from others, concentrated effort is also needed in the Omani educational setting to address the issue of bullying.

## 5. Conclusion

This study corroborates evidence from different parts of the world that bullying among school pupilsis rife and that it is irrespective of gender. The majority of harassment was described to occur within the vicinity of schools. Almost one-third of the victims did not know the reason for the bullying and only a small percentage sought help in the schools after the incident. While bullying has little impact on academic failure, it did have a negative repercussion on attendance. It is therefore imperative that educational environments in Oman are made conducive for learning, particularly considering that the bulk of its population is still within the school-going population.

## Figures and Tables

**Table 1:** Sociodemographic characteristics of the study participants, Oman, 2007.

Characteristics	Total (*N* = 1229)
	*n* (%)
Age (years)	
12	57 (4.6)
13	614 (50)
14	415 (33.8)
>14	143 (11.6)
Gender	
Female	675 (54.9)
Male	554 (45.1)
Area of residence	
As Seeb	391 (31.8)
Muscat/Mutrah	252 (20.5)
Bawshar	284 (23.1)
Al Amerat/Qurayyat	302 (24.6)
Positive history of bullying	
Total	940 (76.5)
Male	422 (76.1)
Female	518 (76.7)

**Table 2:** Characteristics of bullying incidents among school pupils in a 12-month period stratified by gender, Oman, 2007.

Characteristics	Total *n* (%)	Male *n* (%)	Female *n* (%)	*P* value
Frequency of bullying incidents	**929**	**416**	**513**	**0.07**
1–3	671 (72.2)	283 (68)	388 (75.6)	
4–10	121 (13)	61 (14.7)	60 (11.7)	
>10	137 (14.7)	72 (17.3)	65 (12.7)	
Type of bullying	**1951**	**915**	**1036**	**0.04**
Verbal	582 (29.8)	283 (30.9)	299 (28.9)	
Misused	275 (14.1)	121 (13.2)	154 (14.9)	
Physical	534 (27.4)	275 (30.1)	259 (25)	
Social	560 (28.7)	236 (25.8)	324 (31.3)	
Place of bullying	**1549**	**767**	**782**	**0.01**
School	747 (48.2)	328 (42.8)	419 (53.6)	
Neighbourhood	206 (13.3)	158 (20.6)	48 (6.1)	
Home	265 (17.1)	92 (12)	173 (22.1)	
Shopping area	90 (5.8)	48 (6.3)	42 (5.4)	
Bus	241 (15.6)	141 (18.4)	100 (12.8)	

**Table 3:** Characteristics of bullying perpetrators and the reactions of the victims towards bullying incidents among school pupils in a 12-month period stratified by gender, Oman, 2007.

Characteristics	Total *n* (%)	Male *n* (%)	Female *n* (%)	*P* value
Age of perpetrator	**1463**	**688**	**775**	**0.02**
Same age as the victim	382 (26.1)	216 (31.4)	166 (21.4)	
Older than the victim	501 (34.2)	209 (30.4)	292 (37.7)	
Younger than the victim	129 (8.8)	68 (9.9)	61 (7.9)	
Relationship to victim	**1463**	**688**	**775**	**0.04**
A relative of the victim	227 (15.5)	81 (11.8)	146 (18.8)	
Unknown to the victim	224 (15.3)	114 (16.6)	110 (14.2)	
Reasons for bullying	**1020**	**514**	**506**	**0.01**
Physical appearance	266 (26.1)	138 (26.8)	128 (25.3)	
Academic performance	129 (12.6)	79 (15.4)	50 (9.9)	
Manner of speech	113 (11.1)	69 (13.4)	44 (8.7)	
Having a disease or disability	85 (8.3)	58 (11.3)	27 (5.3)	
Unknown	427 (41.9)	170 (33.1)	257 (50.8)	
Informing about bullying	**1431**	**630**	**801**	**0.04**
No reaction	272 (19)	109 (17.3)	163 (20.3)	
Informing a relative	299 (20.9)	110 (17.5)	189 (23.6)	
Informing school staff	171 (11.9)	89 (14.1)	82 (10.2)	
Informing another student	225 (15.7)	68 (10.8)	157 (19.6)	
Reciprocating the bullying	**1431**	**630**	**801**	**0.05**
No reciprocating	967 (67.6)	376 (59.7)	591 (73.8)	
Reciprocating verbally	240 (16.8)	98 (15.6)	142 (17.7)	
Reciprocating physically	224 (15.7)	156 (24.8)	68 (8.5)	

**Table 4:** Comparison between bullied and nonbullied pupils for academic years failed and days of school missed, Oman, 2007.

Outcomes	Total	Bullied	Not bullied	*P* value
Academic years failed (*n*)	**1205**	**923**	**282**	**0.02**
0	1066 (88.5)	828 (89.7)	238 (84.4)	
1	104 (8.6)	74 (8)	30 (10.6)	
2	25 (2.1)	14 (1.5)	11 (3.9)	
3 or more	10 (0.8)	7 (0.8)	3 (1.1)	
Days of school missed (*n*)	**1183**	**547**	**636**	**0.01**
0	327 (27.1)	126 (23)	201 (31.6)	
2-3	591 (49)	265 (48.4)	326 (51.3)	
4–6	190 (15.8)	114 (20.8)	76 (11.9)	
7 or more	75 (6.2)	42 (7.7)	33 (5.2)	
